# Effect of malting period on physicochemical properties, minerals, and phytic acid of finger millet (*Eleusine coracana*) flour varieties

**DOI:** 10.1002/fsn3.696

**Published:** 2018-08-22

**Authors:** Henry O. Udeh, Kwaku G. Duodu, Afam I. O. Jideani

**Affiliations:** ^1^ Department of Food Science and Technology School of Agriculture University of Venda Thohoyandou South Africa; ^2^ Faculty of Natural and Agricultural Sciences Department of Food Science University of Pretoria Hatfield South Africa

**Keywords:** *Eleusine coracana*, ICP‐MS, malting, minerals, phytic acid

## Abstract

**Background:**

Deficiency of essential minerals is a widespread nutritional disorder in the world, particularly in developing economies. Poor mineral accessibility from foods is a major contributing factor to deficiency and associated health problems. This study investigated the effect of malting on minerals, phytic acid, and physicochemical properties of finger millet varieties. Sorghum was used as external reference. Mineral composition was analyzed using an inductively coupled plasma atomic emission spectroscopy (ICP‐AES) and mass spectroscopy (ICP‐MS).

**Results:**

Data showed that finger millet is rich in macroelements and trace elements. Malting for 24 hr reduced mineral content of the grains except sodium. Increase in the minerals was observed beyond 48 hr of malting particularly at 96 hr. Successive decrease in phytic acid of the grains was not observed with malting time. Malting did not result in any significant change in the physicochemical properties of the grains.

**Conclusion:**

ICP‐AES/MS showed that finger millet contain a variety of minerals in amounts that were not previously reported, and malting the grain for 72 to 96 hr positively affected the minerals. Changes in phytic acid suggest that phytate undergoes dissociation during malting rather than a degradation of phytic acid. Potential exists for utilization of finger millet as functional ingredient to augment important minerals in weaning, geriatric, and adult foods for health promotion.

## 1 INTRODUCTION

Finger millet, *Eleusine coracana* (L.) Gaertn, is a small seeded subsistence food crop belonging to the grass family Poaceae. It has other several names, but is more commonly known as *ragi or madua* in India*, rapoko or poho* in Zimbabwe/South Africa, and *dagussa* or *tokuso* in Ethiopia (Kumar et al., 2016; NCR, 2008). Twelve percent (12%) of the global millet area is under finger millet cultivation, reaching more than 25 countries of Africa and Asia (Kumar et al., 2016). Finger millet serves as a staple food to millions of people living in semi‐arid regions of Africa and Asia with low economic status (Reddy, Rao, Reddy, & Kishor, 2008). It has a distinct advantage of being a drought and pest resistant crop, requires little irrigation and other input, and yet sustain optimal yield. Finger millet has been perceived as a potential “super grain” by the United States National Academies as one of the most nutritious among all major cereal grains (Gupta et al., 2017; Kumar et al., 2016; Radchuk et al., 2012; Reddy et al., 2008). However, finger millet is underutilized even in areas of production due to minimal inclusion in ready‐to‐use or ready‐to‐eat convenience food products, unawareness by the general population, lack of research, and novel product development processes (Kumar et al., 2016; Radchuk et al., 2012; Shahidi & Chandrasekara, 2013). The very small size and subsistent scale of production are also a significant technological setback to utilization.

A number of certain inorganic minerals are essential for numerous biological activities which are important for human performance and survival and therefore should be provided in the diet. They constitute a large part of the body intracellular fluid where they act as cofactors to several enzyme systems, neuronal transmission, muscle contraction, and ionic balance. Beside their bodily functions, studies have shown that they play an important role in the treatment of metabolic conditions such as diabetes, hypertension, cardiovascular disorder, anemia, osteoporosis, and immune abnormalities among others (Fraga, 2005; Goldhaber, 2003; Sasaki et al., 1999; World Health Organisation and Food and Agriculture Organisation of the United Nations (WHO/FAO), 2004). Deficiency of essential minerals such as calcium, potassium, magnesium, iron, and zinc are common, and widespread nutritional disorder in the world particularly in developing economies (Platel, Eipeson, & Srinivasan, 2010; WHO, 2001; WHO/FAO, 2004). Another major concern of deficiency of important minerals is in gluten‐free diets. Gluten‐free foods which are an important trend in the nutrition of people who are allergic to gluten or wheat, and in other healthy individuals who follow gluten free foods, have also been shown to contain less valuable minerals than gluten containing food products (Goldhaber, 2003). Poor accessibility of minerals from plant food is a major contributing factor to their deficiency and associated health problems (Fraga, 2005). In cereal grain, particularly, mineral availability is associated with antinutritional factors like phytic acid and polyphenols that form complexes with the minerals thus hindering their release.

Finger millet is considerably rich in minerals, and its micronutrient density is higher than that of major cereal grains: rice and wheat (Kumar et al., 2016). Its calcium and iodine content is reported to be the highest among cereals (Reddy et al., 2008). The nutrient‐rich grain is mainly used for making unleavened bread among other variety of preparations such as cakes, puddings, porridge, etc. The grain malt flour are used in the preparation of infant and geriatric foods, and as a popular food supplement for diabetics (Kumar et al., 2016; Reddy et al., 2008). Finger millet is gluten‐free and hence is good for patients suffering from celiac disease (Pagano, 2006; Saturni, Ferretti, & Bacchetti, 2010). It has been recently used as composite flour for making biscuit due to its high calcium content (Krishnan, Dharmaraj, & Malleshi, 2012). However, due to its high phytate (Afify, El‐Beltagi, El‐Salam, & Omran, 2011; Jha, Krishnan, & Meera, 2015; Mamiro, Van Camp, Mwikya, & Huyghebaert, 2001; Sripriya, Anthony, & Chandra, 1997) and polyphenol (Krishnan et al., 2012; Tako, Reed, Budiman, Hart, & Glahn, 2015) content, the availability of minerals in finger millet is low thus presenting ground for concern. A study by Platel et al. (2010) reported that malting had no significant influence on the mineral content of finger millet except for wheat where a slight decrease in copper content was observed. Mamiro et al. (2001) did not find any significant change in calcium, zinc, and iron content of soaked and germinated finger millet. The report of Sripriya et al. (1997) showed that germination was effective in increasing trace elements such as copper, zinc, and manganese while fermentation was more effective in increasing calcium, phosphorus, and iron from finger millet. Information on the mineral composition of finger millet and how they are affected by malting period and phytic acid content is relatively scarce and inconclusive, and thus requires further elucidation. Here we report the application of inductively coupled plasma atomic emission spectroscopy (ICP‐AES) and mass spectroscopy (ICP‐MS) as a tool to study the minerals present in finger millet grain. Changes in the physicochemical properties and phytic acid content of the grain were also monitored.

## 2 MATERIALS AND METHODS

### 2.1 Cereal grain samples

Local finger millet (brown and dark brown colored) and red‐colored sorghum grain varieties were purchased from retail outlet in Thohoyandou, Limpopo province, South Africa. Megazyme K‐PHYT assay kit was purchased from Megazyme International Ireland, Bray Business Park, Bray Co. Wicklow, A98 YV29, Ireland. All other chemicals used were of analytical grade.

### 2.2 Malting of the grain samples

The cereal grains were malted according to the method of Chethan, Sreerama, and Malleshi (2008). Two varieties of finger millet, namely a brown finger millet (BFM) and dark brown finger millet (DBFM), were used in this study while sorghum was used as the control. Sorghum grain was used as external reference in the study due to the difficulty in obtaining other local varieties of finger millet in the region, and also to validate the technique applied to the test samples. One hundred gram (100 g) portion of the finger millet and sorghum samples was initially soaked in water for 24 hr at 25°C in a growth chamber; the grains were spread on a clean cheese cloth and maintained moist by sprinkling water periodically at intervals of 24 hr. The sprouted grains were kilned for 8 hr at 50°C, after which a characteristic malt aroma was obtained. The kilned grains were milled into fine flour in a stainless steel grinder and stored in polyethylene bags at −20°C until analysis.

### 2.3 Physicochemical properties of the grain samples

The pH of the milled flour was determined according to AOAC (2000) using a pH meter. Color determination was performed using Lovibond spectrocolorimeter (Model LC 100). Upon calibration of the spectrocolorimeter, the unmalted and malted finger millet and sorghum grains were placed in cuvettes and inserted in the device for color measurement.

### 2.4 Determination of mineral content of the grain samples

To solubilize the acid‐extractable elemental content of the samples, digestion was performed on a MARS microwave digestor, using ultra‐pure HNO_3_, or HNO_3_ + HCl at elevated temperature and pressure. After a cooling period, the extractant was made up to 50 ml volume with deionized water, then analyzed by ICP‐AES and/or ICP‐MS for the selected analytes. Major elements were analyzed on a Thermo ICap 6200 ICP‐AES. The instrument was calibrated using NIST (National Institute of Standards and Technology, Gaithersburg, MD, USA) traceable standards to quantify selected elements. A NIST‐traceable quality control standard of a separate supplier than the main calibration standards were analyzed to verify the accuracy of the calibration before sample analysis. Where sample have undergone a digestion step, the results were corrected for the dilution factor resulting from the digestion procedure. Cobalt and selenium were analyzed on an Agilent 7900 quadrupole ICP‐MS. Samples were introduced via a 0.4 ml/min micro‐mist nebulizer into a peltier‐cooled spray chamber at a temperature of 2°C, with a carrier gas flow of 1.05 L/min. The elements were analyzed under He‐collision mode to remove polyatomic interferences.

### 2.5 Phytic acid and total phosphorus content of the grain samples

Phytic acid and total phosphorus content of the unmalted and malted finger millet and sorghum varieties were determined using Megazyme kit (K‐PHYT, Megazyme, Bray, Ireland). Accordingly, 1 g of the grain flour was extracted with 20 ml of 0.66 M HCL for 24 hr at room temperature. An aliquot of the extract was centrifuged at 4470 g for 20 min, and the supernatant was neutralized with 0.5 ml 0.75 M NaOH. Neutralized extract was subjected to enzymatic dephosphorylation for free and total phosphorus. For free phosphorus, 0.05 ml of the neutralized extract was mixed with 0.62 ml of distilled water, 0.20 ml 0.02% (w/v) NaN_3_, pH 5.5 sodium acetate buffer, and then vortexed and incubated at 40°C for 10 min. Afterward, 0.02 ml of distilled water, 0.20 ml MgCl_2_, ZnSO_4_, 0.02% NaN_3_, pH 10.4 buffer was added, vortexed, and incubated at 40°C for another 15 min. The reaction was stopped with 0.30 ml (50%) C_2_HCL_3_O_2_. For total phosphorus: 0.05 ml of the neutralized extract was mixed with 0.60 ml of distilled water, 0.20 ml 0.02% w/v NaN_3_, pH 5.5 sodium acetate buffer, and 0.02 ml phytase enzyme. The mixture was vortexed and incubated at 40°C for 10 min before the addition of 0.2 ml MgCl_2_, ZnSO_4_, 0.02% NaN_3_, pH 10.4 buffer and alkaline phosphatase suspension, vortexed, and incubated at 40°C for 15 min. The reaction was stopped by the addition of 50% 0.30 ml C_2_HCL_3_O_2_. The mixtures were centrifuged at 2860 g for 20 min. Color regent for colorimetric determination of dephosphorylated extract was prepared using 10% w/v ascorbic acid, 1 M) sulfuric acid and 5% w/v ammonium molybdate. One milliliter (1 ml) of dephosphorylated extract (free and total phosphorus) was mixed with 0.5 ml of the color reagent, vortexed, and incubated at 40°C for 1 hr, and absorbance was read at 655 nm. Optical density (OD) for the standard was recorded using the standard solution supplied along with the kit. Total phosphorus and phytic acid contents were calculated as follows:

$$\text{Phosphorus (g/100 g)}=\text{OD}\;\;\text{for}\;\;\text{standard}\times 0.1112\times \Delta A_{\mathrm{phosphorus}}$$

where ΔA_phosphorus_ = Difference between the total phosphorus and free phosphorus

$$\text{Phytic}\;\text{acid (g/100 g)}=\frac{\text{Phosphorus}}{0.282}$$

### 2.6 Statistical analysis

Data obtained were subjected to a one‐way ANOVA by Duncan's multiple comparison test using SPSS version 24.0. The mean of the values were considered to be statistically significant at *p *<* *0.05, and Pearson's correlation analysis test was used to analyze the significance of correlations at *p <* *0.05 and **p <* *0.01.

## 3 RESULTS

### 3.1 Physicochemical properties

**Figure 1:**
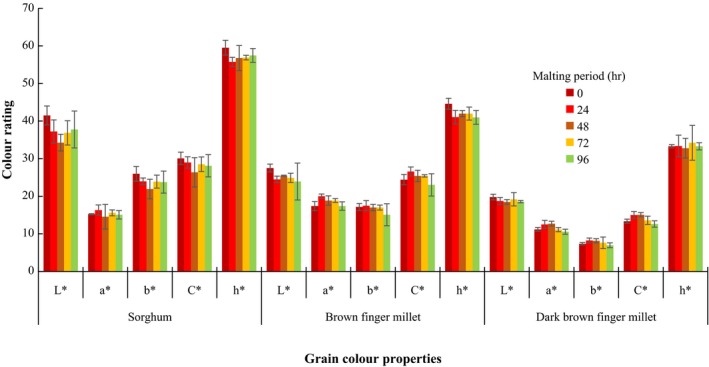
Effect of malting period on the grain color of finger millet and sorghum grains. Values are means of triplicate determinations and are significant at *P *<* *0.05. L*: Positive for whiteness and negative for black; a*: positive for red and negative for green; b*: positive for yellow and negative for blue; C*: Chroma; h*: Hue

The effect of malting period on the pH of the finger millet is presented in Table 1. Malting did not result in a conspicuous decrease in pH of the finger millet varieties and sorghum grain for the entire malting periods. For the grain color properties, the unmalted and malted sorghum grains had higher (*p *<* *0.05) L* values than the corresponding brown and DBFM grains (Figure 1). Lower a* values were observed for unmalted and malted DBFM compared to BFM and sorghum varieties. There was no significant change in yellow color (b*) as a result of malting for DBFM and sorghum grains except for BFM. For chroma (C*) values, changes were observed during malting for DBFM which was lower compared to BFM and sorghum grain. In general, the DBFM had the least color rating compared to BFM and sorghum grains.

**Table 1:** Effect of malting period on the pH, phytic acid, and total phosphorus content of the cereal grains

	Malting period (hr)				
Cereal grains	0	24	48	72	96
	pH				
Dark brown finger millet	6.61 ± 0.01^a y^	6.53 ± 0.02^b x^	6.50 ± 0.01^b z^	6.43 ± 0.02^c z^	6.53 ± 0.02^b z^
Brown finger millet	6.46 ± 0.01^a x^	6.35 ± 0.01^b y^	6.25 ± 0.02^c f^	6.37 ± 0.01^b y^	6.35 ± 0.01^b y^
Sorghum	6.48 ± 0.02^a x^	6.50 ± 0.00^a x^	6.37 ± 0.00^b x^	6.50 ± 0.00^a x^	6.60 ± 0.23^c x^
	Phytic acid (g/100 g)				
Dark brown finger millet	0.3363 ± 0.14^a z^	0.7004 ± 0.00^b y^	0.8352 ± 0.00^c z^	0.7604 ± 0.15^d y^	0.9186 ± 0.14^e y^
Brown finger millet	0.6491 ± 0.00^a y^	0.5271 ± 0.01^b y^	0.7025 ± 0.03^c y^	1.0680 ± 0.01^d y^	0.8645 ± 0.03^e y^
Sorghum	0.2889 ± 0.01^a x^	0.3768 ± 0.26^b x^	0.4535 ± 0.00^c x^	0.3648 ± 0.00^b x^	0.6725 ± 0.00^d x^
	Total phosphorus (g/100 g)				
Dark brown finger millet	0.0948 ± 0.00^a x^	0.1975 ± 0.00^b z^	0.2355 ± 0.00^c z^	0.2144 ± 0.00^d z^	0.2590 ± 0.00^e y^
Brown finger millet	0.1830 ± 0.00^a y^	0.1486 ± 0.00^b y^	0.1981 ± 0.00^c y^	0.3012 ± 0.00^d y^	0.2438 ± 0.01^e y^
Sorghum	0.0815 ± 0.00^a x^	0.1063 ± 0.00^b x^	0.1279 ± 0.00^c x^	0.1029 ± 0.00^b x^	0.1896 ± 0.78^d x^

*Notes*

^abc^Mean within each row for each group not followed by the same superscript are significantly different (*p *<* *0.05).

^xyz^Mean within each column for each group not followed by the same superscript are significantly different (*p *<* *0.05).

### 3.2 Mineral content of the unmalted and malted finger millet and sorghum grain varieties

#### 3.2.1 Boron (B)

**Figure 2:**
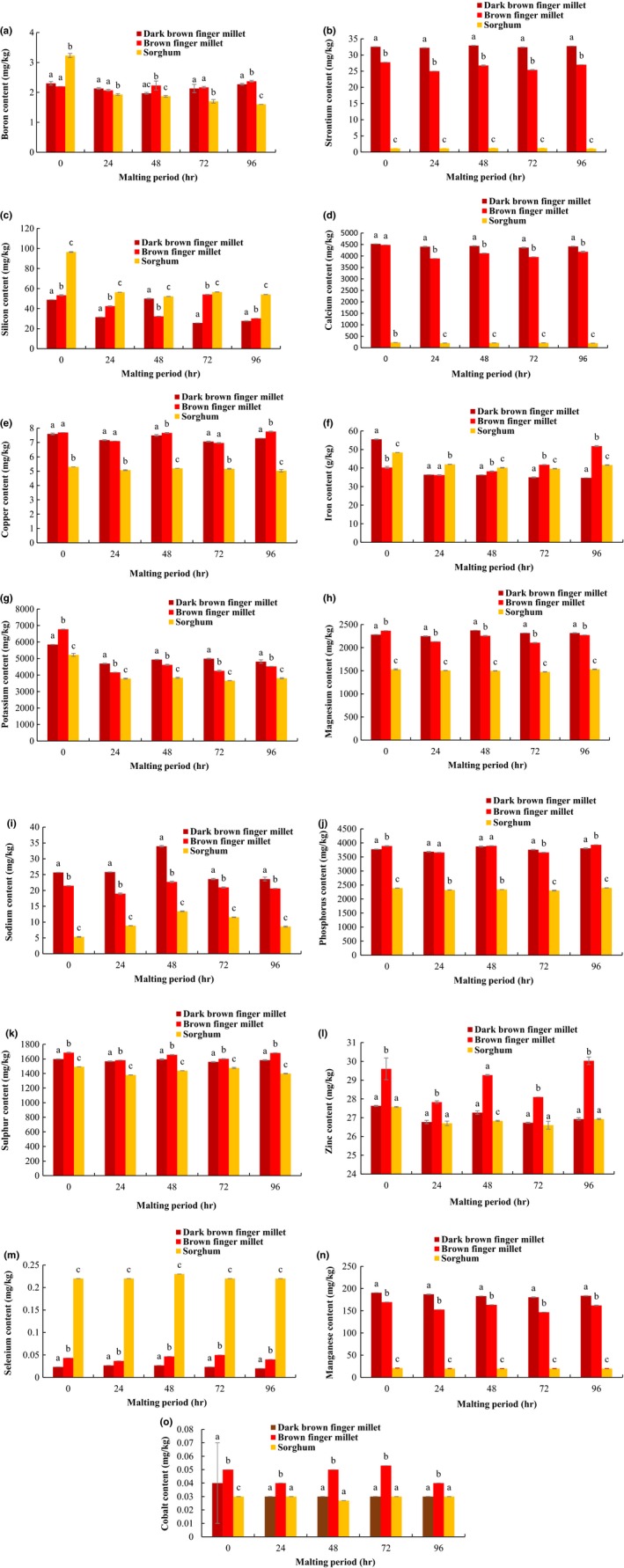
Effect of malting period on mineral content of finger millet and sorghum grains. (a) boron, (b) strontium, (c) silicon, (d) calcium, (e) copper, (f) iron, (g) potassium, (h) magnesium, (i) sodium, (j) phosphorus, (k) sulfur, (l) zinc, (m) selenium, (n) manganese, (o) cobalt. Means ± standard error are significant at *p *<* *0.05

The amount of B from the unmalted and malted food grains are presented in Figure 2a, and they were in the ranges 1.97–2.30 mg/kg in DBFM, 2.07–2.37 mg/kg in BFM and 1.60–3.23 mg/kg in the sorghum grain. The B content of the sorghum grain was significantly (*p *<* *0.05) higher than the finger millet varieties. Unlike the sorghum grain, the finger millet varieties showed insignificant (*p *>* *0.05) decrease in B content during 24 hr of malting. However, at 48 hr of malting, a significant (*p *<* *0.05) decrease in B content of DBFM was observed, which later increased significantly at 72 and 96 hr to levels closer to that in the unmalted counterpart. For the BFM, no significant (*p *<* *0.05) difference in the amount of B was observed within 48 and 72 hr of malting; however, at 96 hr, the B content increased significantly (*p *<* *0.05) close to the amount present in the unmalted grain. Malting for 96 hr brought about a marginal (*p *<* *0.05) decrease in B content of the sorghum grain compared to the finger millet malt varieties.

#### 3.2.2 Strontium (Sr)

The DBFM and BFM varieties were found to contain higher amount of Sr than the sorghum grain, which ranged from 32.20–32.93 mg/kg in DBFM, 25–27.73 mg/kg in BFM and 1.10–1.03 mg/kg in the sorghum grain (Figure 2b). Significant (*p *<* *0.05) decreases in Sr content of the BFM were observed at 24 and 72 hr of malting which later increased, respectively. Although a significant (*p *<* *0.05) increase in the Sr content of BFM was observed at 48 hr, malting at 96 hr resulted in higher Sr content which was close to the amount in the unmalted grain. For the DBFM, a slight (*p *<* *0.05) decrease in Sr content was observed during 24 hr of malting which later increased. Malting for 48 and 96 hr resulted in increases in the Sr content of DBFM which were slightly higher than the amount present in the unmalted grain. However, the amount of Sr content in 48 hr DBFM malt was higher than in the 96 hr malt. For the sorghum grain, significant increase in Sr content was observed at 48 hr which later declined at 96 hr of malting.

#### 3.2.3 Silicon (Si)

The Si content of the unmalted and malted food grains is presented in Figure 2c, and they ranged 25.63–49.97 mg/kg in DBFM, 30.23–53.30 in BFM and 52.13–96.47 mg/kg in the sorghum grain. Significant (*p *<* *0.05) decrease in Si content was observed at 24 hr of malting for the food grains. A further decrease in Si content of BFM and the sorghum grains was observed at 48 hr unlike in DBFM where the malting period resulted in higher amounts of the mineral. Conversely, malting at 72 hr resulted in higher amounts of Si in the BFM and sorghum grains unlike in the DBFM where a significant decrease in the same was observed. Generally, 96‐hr malting resulted in decrease in Si content of the food grains.

#### 3.2.4 Calcium (Ca)

The DBFM and BFM varieties were found to contain higher amount of Ca than the sorghum grain, which ranged 4,408.30–4,524.93 mg/kg in DBFM, 3,888.80–4,483.67 mg/kg in BFM, and 199.43–227.53 mg/kg in the sorghum grain (Figure 2d). Significant decrease in the Ca content of the food grains was observed at 24 hr of malting which later increased. At 96 hr of malting, significantly higher (*p *<* *0.05) amount of calcium was observed for BFM. Whereas for the DBFM, 48 hr of malting resulted in higher amounts of Ca compared 96 hr. Similar observations made for DBFM were recorded for the sorghum malt.

#### 3.2.5 Copper (Cu)

Higher amount of Cu was observed in the finger millet varieties compared to the sorghum grain and was in the range 7.07–7.60 mg/kg in DBFM, 6.97–7.77 mg/kg in BFM, and 5.03–5.30 mg/kg in the sorghum grain (Figure 2e). Reduced (*p *<* *0.05) Cu content was observed during 24 and 72 hr of malting of the millet varieties which later increased, respectively. Increases in the Cu content of BFM were observed at 48 and 96 hr which did not differ significantly from the unmalted grain. Although 96 hr of malting increased Cu content of DBFM, the amount was significantly lower than the amount observed at 48‐hr malt.

#### 3.2.6 Iron (Fe)

The Fe content of the food grains ranged from 34.70 to 55.50 mg/kg in DBFM, 36.20 to 51 mg/kg in BFM, and 39.67 to 48.37 mg/kg in the sorghum grain (Figure 2f). Significant decrease in the Fe content of the food grains was observed at 24 hr of malting which later increased significantly (*p *<* *0.05) with malting time for BFM up to 96 hr. At 96 hr of malting, highest Fe content of 51.83 mg/kg was observed for BFM compared to other malting periods and grain varieties investigated. For DBFM, a slight increase in Fe content was observed at 48 hr which later declined significantly at 96 hr of malting.

#### 3.2.7 Potassium (K)

Compared to other minerals investigated, the food grains were found to contain higher amount of K which ranged 4,698.80–5,850.93 mg/kg in DBFM, 4,161.73–6,775.03 in BFM, and 3,659.63–5,223.50 mg/kg in the sorghum grain (Figure 2g). Significant (*p *<* *0.05) decrease in K content of the food grains was observed at 24 hr of malting thereafter an increase which remained constant up to 96 hr of the sorghum grain. Significantly (*p *<* *0.05) higher amount of K was observed at 48 hr malt of the finger millet varieties which did not differ significantly from the amount observed at 72 and 96 hr the DBFM malt. Although a significant increase in K content was observed at 96‐hr malt of BFM, it was lower than the amount recorded at 48 hr.

#### 3.2.8 Magnesium (Mg)

The Mg content of the unmalted and malted food grains is shown in Figure 2h, and the amount ranged 2,250–2,374.37 mg/kg in DBFM, 2,106.37–2,272.93 mg/kg in BFM, and 1,476.83–1,531.93 mg/kg in the sorghum grain. Significant (*p *<* *0.05) decreases in Mg content of the food grains were observed at 24 and 72 hr of malting. At 96 hr of malting, significantly (*p *<* *0.05) higher amount of Mg was observed for BFM and the sorghum grain, whereas the same was higher at 48‐hr DBFM malt.

#### 3.2.9 Sodium (Na)

The Na content of the food grains ranged 23.60–33.97 mg/kg in DBFM, 18.97–22.70 mg/kg in BFM, and 5.33–13.40 mg/kg in the sorghum grain (Figure 2i). Malting for 48 hr resulted in higher (*p *<* *0.05) amount of Na content of the food grains, which later decreased significantly and remained constant up to 96 hr for the finger millet varieties. For the sorghum grain, however, a further decrease was observed after 72 hr. Unlike other minerals investigated, 24 hr of malting resulted in an increase in the Na content of the BFM and sorghum varieties with a statistically insignificant increase in the same for DBFM.

#### 3.2.10 Phosphorus (P)

The unmalted and malted finger millet varieties and sorghum grain were found to contain high amounts of P which ranged 3,871.40–3,871.40 mg/kg in DBFM, 3,657.83–3,930.10 mg/kg in BFM, and 2,303.27–2,395.40 mg/kg in the sorghum grain (Figure 2j). Significant (*p *<* *0.05) decrease in P content of the food grains was observed at 24 hr of malting thereafter an increase. Malting for 96 hr resulted in significantly (*p *<* *0.05) higher amount of P for the BFM and sorghum varieties. While for DBFM highest amount of P was observed at 48 hr.

#### 3.2.11 Sulfur (S)

The S content of the unmalted and malted food grains ranged 1,559.13–1,597.37 mg/kg in DBFM, 1,582.27–1,686.60 mg/kg in BFM, and 1,380.60–1,492.07 mg/kg in the sorghum grain (Figure 2k). Significant (*p *<* *0.05) decrease in the S content of the food grains was observed at 24 hr of malting which later increased. At 96 hr of malting higher amount of S was observed for BFM, whereas for DBFM, the same was higher at 48 hr which was not significantly different from the amount observed at 96 hr. Malting sorghum grain for 72 hr resulted in higher amounts of S compared to 48 and 96 hr.

#### 3.2.12 Zinc (Zn)

The amount of extractable zinc in the unmalted and malted food grains ranged 26.77–27.63 mg/kg in DBFM, 28.10–30.03 mg/kg in BFM, and 26.60–27.57 mg/kg in the sorghum grain (Figure 2l). Reduced (*p *<* *0.05) Zn content was observed at 24 hr of malting for the food grains thereafter an increase which remained constant up to 96 hr of the sorghum grain. Malting for 96 hr resulted in higher amounts of Zn in BFM. For DBFM, higher amount Zn was recorded at 48 hr which later decreased and remained constant up to 96 hr.

#### 3.2.13 Selenium (Se)

Similar to the Co, lower amount of Se was found in the food grains and it ranged 0.02–0.037 in DBFM, 0.037–0.05 mg/kg in BFM, and 0.22–0.23 mg/kg in the sorghum grain (Figure 2m). The sorghum grain contained higher amount of Se compared to the finger millet varieties. Malting process did not result in any significant change in Se content of DBFM and the sorghum grain. However, for BFM significant (*p *<* *0.05) decrease in Se content was observed at 24 hr of malting. Statistically significant (*p *<* *0.05) increases were observed at 48 and 72 hr of malting which were higher than the amount in the unmalted grain. Malting for 96 hr significantly reduced the amount of Se in compared to 48‐ and 72‐hr BFM malt.

#### 3.2.14 Manganese (Mn)

The amount of Mn present in the unmalted and malted food grains ranged 180.50–190.63 mg/kg in DBFM, 146.73–169.43 mg/kg in BFM, and 19.93–20.97 mg/kg in the sorghum grain (Figure 2n). Higher Mn content was observed for the finger millet varieties compared to the sorghum grain. Significant (*p *<* *0.05) decrease in the Mn content of the food grains was observed at 24 hr of malting which remained constant up to 96 hr of the sorghum grain. For the DBFM, higher amount of Mn was observed at 96 hr which was not significantly different from the 48 hr malt. For BFM, higher amount of Mn was observed at 48 hr of malting compared to other malting periods.

#### 3.2.15 Cobalt (Co)

The food grains were found to contain relatively lower amounts of Co (Figure 2o). Malting did not affect the amount of Co in the sorghum grain. However, it resulted in changes in the finger millet varieties and was in the range 0.03–0.04 mg/kg in DBFM and 0.04–0.05 mg/kg in the BFM. An initial decrease (*p *<* *0.05) in Co content was observed at 24 hr for the DBFM which remained constant for the entire malting period. A similar initial decrease was observed for BFM after which there was an increase. Higher amount of Co was recorded at 48 and 72 hr.

### 3.3 Phytic acid and total phosphorus content

The phytic acid content of the unmalted and malted food grains ranged 0.3363–0.9186 g/100 g in DBFM, 0.5271–1.0680 g/100 g in BFM, and 0.2889–0.6725 g/100 g in the sorghum grain (Table 1). Malting for 24 hr resulted in a significant (*p *<* *0.05) decrease in the phytic acid content of BFM unlike for DBFM and sorghum grain malt. Significant (*p *<* *0.05) increase in the phytic acid content of the cereal grain malt was observed at 48 hr of malting. A further increase was observed at 72 hr of malting for the BFM, whereas the same was reduced significantly (*p *<* *0.05) for the DBFM and sorghum grain malt. Conversely, at 96 hr of malting reduced phytic acid content was observed for BFM, whereas the same was increased for DBFM and sorghum grain malt.

The total phosphorus content of DBFM, BFM, and sorghum grain was in the ranges 0.0948–0.2590, 0.1486–0.3012, and 0.0815–0.1896 g/100 g, respectively (Table 1). Unlike the BFM, malting for 24 hr significantly (*p *<* *0.05) increased the total phosphorus content for DBFM and sorghum grain. A further increase in the total phosphorus content was observed at 48 hr for DBFM and sorghum grain malt which also increased the same for BFM. At 72 hr of malting, reduced total phosphorus content was observed for DBFM and the sorghum grain malt, whereas the same increased significantly for BFM. Similar trend observed at 96 hr of malting for phytic acid content was also recorded for total phosphorus for the cereal grains. In general, the malting process had a similar effect on the phytic acid and total phosphorus content of the individual grain malt.

## 4 DISCUSSION

The physicochemical properties of the finger millet and sorghum varieties were not strongly affected by the malting process, particularly the pH which became slightly acidic. The changes in the color properties of the finger millet and sorghum grain varieties may have resulted from the distinct color types of the cereal grains, which were slightly modified during the malting.

Finger millet and sorghum grain varieties contain a number of nutritionally essential macro‐ and microelements and were significantly affected by malting. The amount of calcium, phosphorus, potassium, magnesium, iron, zinc, manganese, copper, and sodium obtained in this study is comparable with the results of Platel et al. (2010), Sripriya et al. (1997), and Shashi, Sharan, Hittalamani, Shankar, and Nagarathna (2007). Higher amounts of calcium (388.9–452.5 mg/100 g), phosphorus (365.8–393.10 mg/100 g), magnesium (211.0–237.4 mg/100 g), zinc (2.68–3.0 mg/100 g), manganese (14.7–19.1 mg/100 g), copper (0.7–0.78 mg/100 g), and sodium (1.9–3.4 mg/100 g), respectively, were observed in our study compared to the value ranges reported by other researchers. The observation could be as a result of the technique and instrument of determination applied, wherein a combination of nitric and hydrochloric acid was used in the extraction of the minerals unlike in other studies where hydrochloric acid is the main extraction solvent. Also, high amount of sulfur (158.4–168.7 mg/100 g) was observed in the finger millet varieties followed by strontium (2.5–3.3 mg/100 g), silicon (2.6–5.4 mg/100 g) and boron (0.2–0.24 mg/100 g) while cobalt (0.003–0.005 mg/100 g) and selenium (0.002–0.005 mg/100 g) were found at very little amounts. The initial 24‐hr malting resulted in a decrease in the minerals except for sodium. Loss in sodium content was only observed with malting time. Similar observation has been reported by other researchers (Afify et al., 2011; Krishnan et al., 2012; Malleshi & Desikachar, 1986). As biosynthesis or degradation of minerals is not expected during malting, the loss of the minerals may be due to leaching during soaking and germination stages of the food grains. The change could also may have resulted from loss through utilization of other nutrients by the growing embryo. The increase observed for the sodium content during 24 hr of malting could be due to its highly water soluble nature and single oxidation state which potentiates its early release compared to other minerals.

Relevant changes were noted in the mineral composition of the food grains at 48 up to 96 hr of malting. The effect of malting on phytate–mineral complexes has been well established. Finger millet contain high amount of phytate (417 mg/100 g) compared to other cereals such as sorghum (295 mg/100 g), barley (278 mg/100 g), rice (160 mg/100 g), and maize (414 mg/100 g) (Hemalatha, Platel, & Srinivasan, 2007; Sripriya et al., 1997). Several processing techniques have been shown to reduce the amount of phytate in cereal grains, especially malting, wherein phytase, a phytate‐specific enzyme is activated, resulting in the dephosphorylation of inositol phosphates or phytate forms (Luo, Xie, Jin, Wang, & He, 2014). This process often result in phytate complex dissociation and or reconfiguration with principal products being inorganic phosphates, inositol phosphate monomers, divalent and trivalent mineral ions, proteins, and amino acids. Enzymatic hydrolysis of phytate in finger millet during germination was observed by Mbithi‐Mwikya, Van Camp, Yiru, and Huyghebaert (2000) wherein phytate values decreased from 0.35 in the raw sample to 0.02 g/100 g after 96 hr of germination. Mamiro et al. (2001) have shown that 48‐hr germination reduced 49.2% of the total phytic acid content of finger millet. In another report, germination for 72 hr was found to significantly reduce the phytate content in pearl millet compared to 48 hr germinated (Sehga & Kawatra, 1998).

At 48 hr of malting significant increase in iron, magnesium, phosphorus, sulfur, and zinc were observed for DBFM, whereas in BFM and the sorghum grain, the same was found to increase significantly at 96 hr. At 48 hr, the strontium content of the food grains was higher compared to other malting periods. Also, 48 hr of malting resulted in the increased silicon content of DBFM which reduced the same in BFM and sorghum grain. However, at 72 and 96 hr, respectively, the silicon was found in higher amounts in BFM and sorghum grain. Manganese content was higher at 48 hr of malting for BFM, whereas the same was highest for DBFM at 96 hr. Calcium, copper, and boron were found at a significantly higher amount at 96 hr of malting in the finger millet varieties unlike in the sorghum where higher amounts of the same were observed at 48 hr. Selenium and cobalt content were relatively low of which malting did not result in practical change in their amounts. The increase in the mineral content of the cereal grains observed after 24 hr of malting could be due to an accumulative effect resulting from the dissociation and or reconfiguration of phytate complexes and other mineral binding components of the grain during malting, and as well as the presence of mineral enhancers like ascorbic acids (Krishnan et al., 2012; Mamiro et al., 2001; Sripriya et al., 1997). A varietal difference which is a dependent factor in the degradation of phytate complexes was also found to play a major role in the amount of extractable minerals from the cereal grains. The differences in extractable minerals from the grains, particularly at 48 and 96 hr of malting, could be seen as a function of varietal differences. These results are in contrast with the reports of Platel et al. (2010) and Mamiro et al. (2001) who found no significant effect of malting in the mineral content of finger millet.

The phytic acid content of finger millet and sorghum grain varieties obtained in this study is comparable with the report of Hemalatha et al. (2007) who found 417 mg/100 g (0.417 g/100 g) and 295 mg/100 g (0.295 g/100 g) for finger millet and sorghum grains, respectively. The result is comparable with the report of Mbithi‐Mwikya et al. (2000) and Sripriya et al. (1997) who found 0.35 g/100 g and 0.6 g/100 g, respectively. The present result showed that malting had a variable effect on the phytic acid content of the cereal grain varieties as previously described. A malting time‐dependent decrease in phytic acid of the finger millet grains was not observed. Decreases in the phytic acid content of the cereal grains were observed, particularly at 24, 72, and 96 hr of malting which varied for the finger millet varieties and sorghum grain malt. Conversely, increase in phytic acid content was observed at 48 and 96 hr of malting which also varied for the grain malt flour. This observation is contrary to the report of Mbithi‐Mwikya et al. (2000) who showed a steady decrease in phytate content of 0.35 g/z 100 g in the raw sample to 0.02 g/100 g after 96 hr of germination. The discrepancy could arise as a result of the difficulty in estimating accurately the phytic acid content of processed cereal grains which can contain high amounts of other *myo*‐inositol forms (i.e., IP_3_, IP_4_, and IP_5_) that would co‐elute with phytic acid (IP_6_) and contribute to the total phytic acid content. This is not the case for unprocessed grain where phytic acid comprise at least 97% of the total inositol phosphates, hence the coherence in phytic acid content of the unmalted cereal grain with the report of other researchers.

Phytic acid is present in cereal grains in the form of phytate complexes. During malting, phytases are induced which act on both high and low phytates. Principally, phytase reduces the hexa form of phytic acid (IP6, *myo*‐inositol 1,2,3,4,5,6‐hexakisphosphate) into lower forms such as IP5, IP4, IP3, IP2, IP1, and *myo*‐inositol (Agte, Gokhale, & Chiplonkar, 1997). In the process, lower phytic acids IP5, IP4, and IP3 are simultaneously being formed as products from the dissociation and or reconfiguration of other higher inositol phosphates already present like IP5 and IP4 (Qvirist, Carlsson, & Andlid, 2015). This sequence of enzymatic reaction in turn increases the amount of inorganic phosphate and lower phytic acids, which co‐elute as total phytic acid content. It is suggested that the differential effect of malting on the phytic acid content of the grains could be as a result of the combined formation and dissociation of intermediate inositol phosphates alongside the principal form of phytic acid during the malting periods. Unlike the phytic acid content, the malting periods virtually resulted in a malting time‐dependent increase in phosphorus content of the cereal grain malt flours. The observation could be as a result of inorganic phosphorus released during malting through hydrolysis of lower inositol phosphates together with the principal form of phytic acid (IP6).

Pearson's correlation coefficients between the minerals, phytic acid, total phosphorus, and pH of the finger millet and sorghum grain malt are presented in Table 2. A significant positive correlation (*p *<* *0.05) was observed between phytic acid, calcium, magnesium, potassium, manganese, and copper for the malting periods particular at 24 and 72 hr, suggesting that changes in phytate content of the grain malt contributed to the release of the minerals. No significant correlation (*p *>* *0.05) was observed between phytic acid, selenium, and boron. This could be as a result of the very minute amounts in which they occur in the finger millet grain. Significant negative correlations between phytic acid, total phosphorus, and zinc were observed. Among the minerals, significant positive and negative correlation coefficients were recorded particularly for cobalt, copper and manganese where negative correlation was observed. Apparently, there are limited studies that have described the interaction between minerals, and phytic acid content of finger millet. Sripriya et al. (1997) observed a decreased phytate/Zn molar ratio from 19.2 to 7.8 for 48‐hr germinated finger millet, which suggests a negative interaction of phytic acid and zinc availability as observed in the present study.

**Table 2:** Pearson's correlation coefficients between phytic acid, total phosphorus, power of hydrogen, and minerals of the finger millet and sorghum varieties

Response variables	TP	pH	Ca	Mg	K	Zn	Cu	Mn	P	S	Na	Si	Co	Se	Sr	B	Fe
PHYT	−0.999*	−0.998*	1.000*	0.998*	0.999*	−1.000*	1.000*	1.000*	−0.938*	1.000*	1.000*	0.999*	−0.944	0.982	1.000**	0.971	−.0835
TP		−0.998*	1.000*	0.998*	0.999*	−0.998*	−0.603	1.000**	1.000*	1.000*	1.000**	0.999*	−0.943	0.982	−0.982	0.944	−0.835
pH			0.929	0.998*	0.999*	1.000**	0.866	−1.000**	0.968	0.964	0.968	0.910	1.000**	−0.929	−1.000**	−0.982	0.990
Ca				1.000*	0.997*	1.000*	0.980	1.000**	1.000*	0.999*	0.999*	0.998*	−0.965	0.956	0.995	1.000**	0.999*
Mg					1.000**	0.999*	1.000**	0.998*	1.000**	1.000**	1.000**	0.998*	−0.963	0.971	0.999*	0.963	1.000**
K						0.964	0.960	0.999*	1.000**	0.999*	1.000*	0.999*	0.957	0.909	0.999*	0.960	1.000*
Zn							−0.500	1.000*	0.999*	1.000*	0.961	1.000*	1.000**	−0.500	0.945	1.000*	1.000*
Cu								0.945	0.976	0.969	1.000**	1.000*	−1.000**	1.000**	0.866	1.000**	1.000**
Mn									1.000**	0.999*	1.000**	1.000*	−1.000**	−0.715	0.992	1.000**	0.999*
P										1.000**	0.999*	0.999*	−0.961	−0.806	0.998*	1.000**	0.999*
S											0.999*	0.999*	−0.956	−0.945	1.000*	1.000*	1.000*
Na												1.000*	−0.945	−0.866	1.000**	0.866	1.000*
Si													−0.945	−1.000**	0.982	0.993	0.997*
Co														0.945	−0.866	1.000**	−0.277
Se															−0.866	−1.000**	−0.866
Sr																0.993	1.000**
B																	0.999*

*Note*

Pearson correlation coefficients *, ** indicate significance at *p *<* *0.05 and 0.01. PHYT: phytic acid; TP: total phosphorus; pH: power of hydrogen; Ca: calcium; Mg: magnesium; K: potassium; Zn: zinc; Cu: copper; Mn: manganese; P: phosphorus; S: sulfur; Na: sodium; Si: silicon; Co: cobalt; Se: selenium; Sr: strontium; B: boron; Fe: iron.

The pH of plant food plays an important role in availability and/or bioavailability of minerals both in food material and during digestion in the human gut. Under acidic conditions, transition/dissociation of mineral into their ionic state occurs which result in their precipitation under increasing pH, thereby limiting their bioavailability (Skibsted, 2016). In addition, pH affects the hydrolysis of phytate and organic acid complexes which are important for mineral accessibility in cereal grains. The present study showed a significant negative correlation (*p *<* *0.05) between pH and phytate content of the malted grains, thus suggesting that increase in acidity contribute to changes in phytate that affects mineral availability in finger millet.

## 5 CONCLUSION

Application of ICP‐AES/MS reveals that finger millet is a rich source of both macroelements and trace elements in amounts that were not previously reported, and that malting for 72–96 hr positively influenced certain minerals of the grain. Malting for 48 and 96 hr had a better impact on the mineral composition which varied for the grain varieties. The majority of the minerals including magnesium, calcium, phosphorus, iron, zinc, and copper were enhanced at 96 hr of malting for BFM, whereas for DBFM, the reverse was the case except for manganese and strontium. Selenium and cobalt content of the grains were relatively low and were not affected by malting. The changes in phytic acid content of the grain malt suggest that phytate, the principal form of phytic acid present in grains, undergoes dissociation and/or reconfiguration during malting rather than a degradation of phytic acid. Varietal difference was found to play an important role on how processing method affects the minerals and phytic acid content of the grain. This study provides a rationale for increased utilization of finger millet grain as a functional food ingredient, for the alleviation of mineral deficiencies in children and adult foods.

## CONFLICT OF INTEREST

The authors declare that there are no conflicts of interest.
